# Transesophageal echocardiography guidance for percutaneous closure of PFO and a new method to improve the diagnosis and safety during the procedures

**DOI:** 10.3389/fcvm.2024.1428380

**Published:** 2024-07-31

**Authors:** Limin Luo, Zehan Xie, Qiaoyan Wu, Qiang Liu, Huiping Hou, Yongshi Wang, Xianhong Shu

**Affiliations:** ^1^ Department of Echocardiography, Zhongshan Hospital (Xiamen), Fudan University, Xiamen, Fujian, China; ^2^ Department of Echocardiography, Zhongshan Hospital, Fudan University, Shanghai, China

**Keywords:** echocardiography, transesophageal echocardiography, PFO, percutaneous PFO closure, cryptogenic stroke, migraine atrial septal defect, cerebral infarction (CI), cryptogenic stroke (CS)

## Abstract

**Purpose:**

Percutaneous patent foramen ovale (PFO) closure is becoming more and more common for the treatment or prevention of PFO-associated right-to-left shunt (RLS). This study aims to investigate the value of transesophageal echocardiography (TEE) in percutaneous PFO closure, and to explore a new method that can improve intraoperative diagnosis and surgical safety.

**Materials and methods:**

Based on our inclusion and exclusion criteria, we enrolled 73 patients between 16 and 70 years old (average age 43.25 ± 14.87 years) who underwent percutaneous PFO closure at the Department of Cardiac Surgery, Zhongshan Hospital (Xiamen), Fudan University, from January 2022 to December 2023. Out of the 73 enrolled patients, there were 28 males (38.36%) and 45 females (61.64%), 29 migraine patients (39.73%), 14 patients (19.19%) with headache and dizziness, 14 patients (19.18%) with a history of cerebral infarction (CI), and 25 patients (34.25%) with CI, lacunar infarction or ischemic focus on magnetic resonance imaging (MRI). All patients received routine transthoracic echocardiography (TTE) and agitated saline contrast echocardiography (ASCE) before operations. Percutaneous closure of PFO was completed under the guidance of TEE. In 12 patients, the method of “injection of heparinized sterile saline through the delivery sheath” was used to observe their RLS, and the anatomical characteristics of the PFO according to the shunt path were monitored and evaluated. This method was also applied to some patients to guide the conveyor to pass through the foramen ovale (FO) channel safely and effectively, thereby improving the success rate of PFO closure.

**Results:**

The application of TEE during the procedure of percutaneous PFO closure, including preoperative evaluation, intraoperative guidance, and postoperative reevaluation, can offer further details about the anatomical and shunt characteristics of PFO, improve the diagnosis rate, and confirm the safety of the surgical path. It ensures the safety and reliability of the whole operation, greatly improving the success rate and reducing postoperative complications.

**Conclusions:**

TEE guidance of percutaneous PFO closure has the advantages of minimal trauma, no radiation and real-time visualization, while injecting heparinized sterile saline through the delivery sheath is safer and more effective in improving the success rate and reducing postoperative complications.

## 1 Introduction

Fossa ovale (FO) is the pore located between the septum primary and secondary septum during fetal development, an important structure to maintain normal fetal circulation. After birth, as a baby cries and the pleural pressure drops, the left atrial (LA) pressure increases, pushing the primary septum to the secondary septum and making the two septums adhere and fuse. This process gradually forms a permanent atrial septum. Failing to complete this process leads to an interatrial slit-like channel called patent foramen ovale (PFO) (1, 2). The global prevalence of PFO is approximately 27% (2). An autopsy study showed the incidence of PFO was 30% in the age group of 1–29 years, 25% in the age group of 30–79 years, and 20.2% in the age group of over 80 years (3). As a common congenital cardiac abnormality, the incidence of PFO ranges from 20% to 34% (4). In general, the primary septum is thin and long, and the secondary septum is thick and muscular. Under normal circumstances, because the LA pressure is higher than the right atrial (RA) pressure, the primary septum and secondary septum are closely attached, and the FO is in a closed state. The FO may remain persistently open in the presence of a PFO or may open when the thin, long primary septum is pushed apart as the RA pressure becomes higher than LA (e.g., coughing, sneezing, or constipation). Blood, thrombus, or any substance in the blood (including vasoactive substances such as 6-tibiottryptamine and platelet-derived factor) from the right heart system can enter the arterial system through the open FO to form an embolism (paradoxical embolism). The long-standing concept of PFO is that it has no special clinical significance, so it has not been paid much attention. In fact, however, PFO is associated with a variety of clinical symptoms, including ischemic stroke, transient ischemic attack (TIA), migraine, systemic or coronary embolism, and so on. PFO closure is a way to alleviate or prevent the related symptoms, especially for those young patients (under 60 years old) who have had cerebral infarction and PFO complications. When other causes of cerebral infarction are excluded, PFO closure plays an important role in successfully preventing the recurrence of cerebral infarction.

At present, percutaneous PFO closure can be performed under the guidance of x-ray or TEE-guided ultrasound. While many of the procedures are still under the guidance of x-ray, the TEE-guided percutaneous PFO closure is more widely used in recent years. This study aims to investigate the value of TEE in percutaneous closure of PFO, and to explore a new method that can be used to improve the intraoperative diagnosis and safety of the operation.

## 2 Material and methods

### 2.1 Patient population and study design

We enrolled 73 patients between 16 and 70 years old (average age 43.25 ± 14.87 years) who underwent percutaneous PFO closure at the Department of Cardiac Surgery, Zhongshan Hospital (Xiamen), Fudan University, from January 2022 to December 2023, including 28 males (38.36%) and 45 females (61.64%), 29 patients with migraine (39.73%),14 patients (19.19%) with headache and dizziness, 14 patients (19.18%) with a history of cerebral infarction, 25 patients (34.25%) with cerebral infarction, lacunar infarction or ischemic focus on MRI, 21 patients (28.77%) with PFO detected by TTE color flow Doppler presenting left-to-right shunt (LRS) at the FO, 66 patients (90.41%) with PFO confirmed by TEE before admission(there are both gaps and shunts), and 7 patients (9.59%) with PFO showing a small gap yet inevident shunt by TEE before admission but right-to-left shunt (RLS) suggested by ASCE. According to the 2019 SCAI guidelines (5), although some patients did not have strong indications for PFO closure, they were also clinically included in the cohort because of the strong demand of these patients or their families. All patients had significant clinical symptoms, including migraine, dizziness or vertigo, and syncope that could not be controlled by medications. Some patients experienced these symptoms accompanied by nausea and vomiting, while severe patients were accompanied by loss of consciousness—the most serious was cerebral infarction. The course of the disease ranged from 1 month to 40 years, and other causes that could cause related symptoms or cerebral infarction were excluded before surgery.

**Inclusion criteria:**

1. Age between 16 and 70 years old, including 16 and 70 years old;
2. Combined PFO and clinically excluded other causes of related diseases: (1) cryptogenic stroke (CS) or transient ischemic attack (TIA); (2) refractory migraine with no response to medications;
3. Large PFO (channel inner >4 mm);
4. Complex PFO: PFO with multiple shunts; long channel PFO (≥8 mm); combined with atrial septal aneurysm (ASA); accompanied by a lengthy Eustachian valve; hypertrophic secondary atrial septum (>10 mm), and with complicated atrial septal defect (ASD);
5. Patients or their authorized proxy agree to have the PFO closure performed under general anesthesia.
6. Patients or their authorized proxy agreed to sign the informed consent before surgery.

**Exclusion criteria**:

1. Patients with other cardiac diseases requiring surgical treatment;
2. Patients combined with esophageal stenosis or perforation, esophagotracheal fistula, active esophageal or gastric bleeding and other patients who are not suitable for TEE monitoring.

### 2.2 Methods

#### 2.2.1 TTE

TTE was conducted using a Philips EPIQ 7C ultrasound system equipped with an S5-1 1–5 MHz transducer (Philips Ultrasound, Holland). All patients underwent a complete TTE examination by an experienced sonographer. Patients were scanned in the left lateral decubitus position and connected to an electrocardiogram. All two-dimensional (2D) and Doppler recordings and measurements were performed according to American Society of Echocardiography guidelines (6). We mainly observed the atrial septum in the parasternal aortic short-axis view, parasternal 4-chamber view, and subcostal biatrial view. The presence of PFO was considered if two or more views confirmed a thin bundle of oblique LRS at the FO. In addition to focusing on the PFO, it is also important to rule out the presence of other structural lesions, the presence of thrombosis, and other intracardiac diseases that may be related to the clinical symptoms, because it will affect the clinical management decisions.

#### 2.2.2 ASCE

ASCE was also performed by an experienced sonographer using a Philips EPIQ 7C ultrasound system equipped with an S5-1 1–5 MHz transducer (Philips Ultrasound, Holland). Patients were also scanned in the left lateral decubitus position and connected to an electrocardiogram. Every patient was cannulated in the right antecubital with an 18-gauge cannula. The cannula was connected with an extension tube, to which is connected a three-way tap and two ten 10 ml syringes, respectively 8 ml of sterile saline, one 1 ml of air and 1 ml of the patient's blood. They were thoroughly exchanged at least 10 times to produce an air suspension. During this time the patient was asked to do the Valsalva maneuver (VM) for around 10 s. If the VM was effective we can find that the interatrial septum was pushed to the LA by the improving pressure of the RA. Then, the air suspension was injected into the patient immediately. When the micro-bubble (MB) can be seen in the RA, the patient was asked to exhale quickly. If any MB was observed in the LA immediately or in 3–5 cardiac cycles, PFO was diagnosed.

#### 2.2.3 TEE

TEE were performed by using a Philips EPIQ 7C ultrasound system equipped with an X7-2t 2-7MHz transducer (Philips Ultrasound, Holland). If there was a “slit-like” channel between the primary septum and secondary septum and color Doppler showed LRS, PFO was confirmed. Because the shunt was impacted by the pressure between left and right atrial, sometimes the shunt was not obvious. In this case, the patient's medical history and the result of the ASCE may be important to the diagnosis of PFO.

### 2.3 Intraoperative TEE-guided percutaneous PFO closure

**Figure 1:**
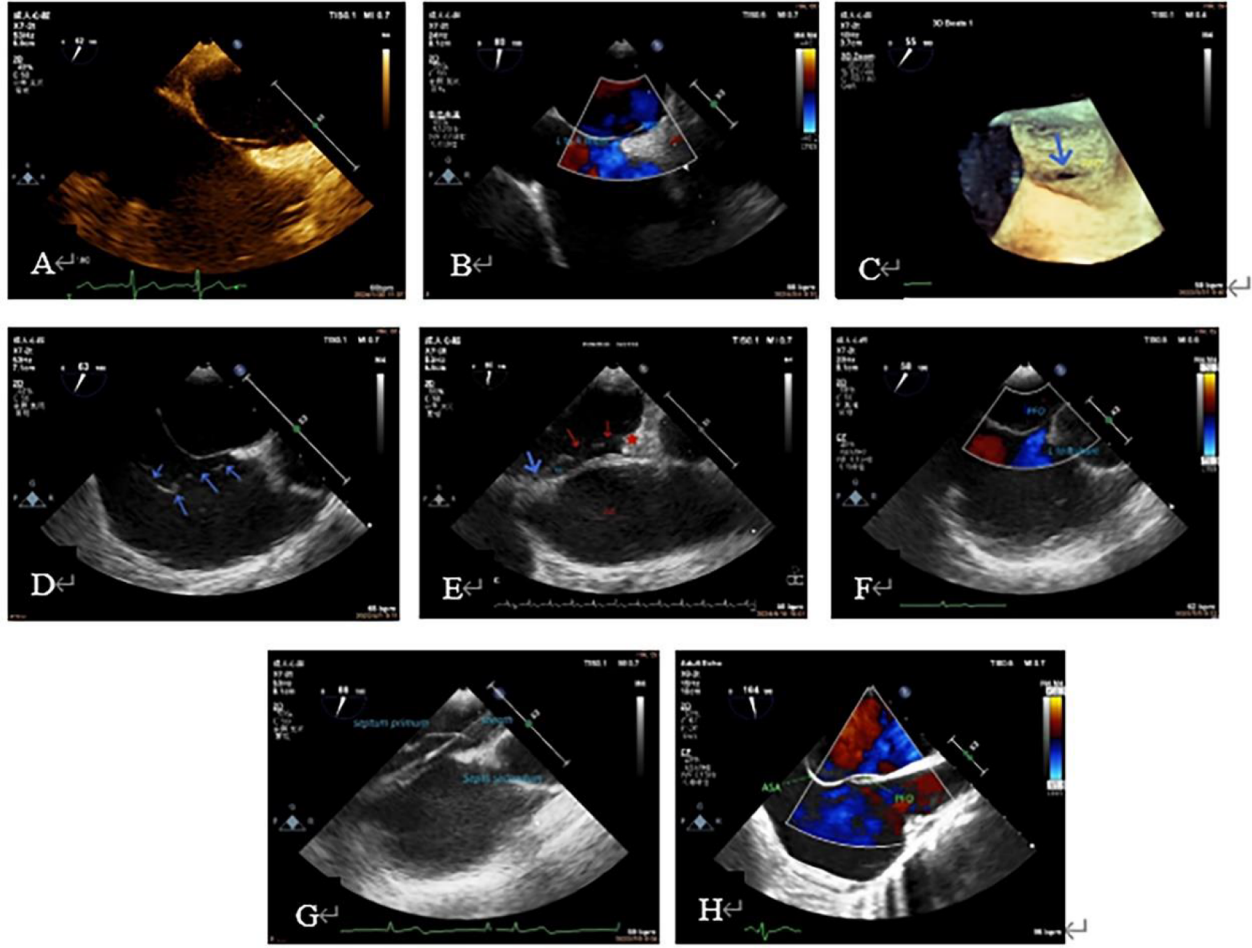
Preoperative TEE to understand PFO shape, size, shunt, thickness and mobility of the primary septum, whether it is combined with ASA, excessive eustachian valve, and whether it is combined with other structural problems that need to be treated. (**A**): PFO tunnel showed a small RA opening and a large LA opening. (**B**): LRS of PFO; (**C**): PFO under 3D ultrasound (blue arrow); (**D**): excessive Eustachian valve; (**E**): the primary septum of the PFO (shown by blue arrow) was connected with the coumadin ridge (shown by red star). The PFO was continuously open, and two continuous interruptions were observed on the primary septum (shown by red arrow). (**F**): Preoperative diagnosis of ASD, intraoperative TEE showed a large PFO; (**G**): primary septum with large mobility. (**H**): TEE showe ASA. 3D, three-dimensional; ASA, atrial septal aneurysm; ASD, atrial septal defect; LA, left atrial; LRS, left-to-right shunt; PFO, patent foramen ovale; RA, right atrial; TEE, transesophageal echocardiography.

**Figure 2:**
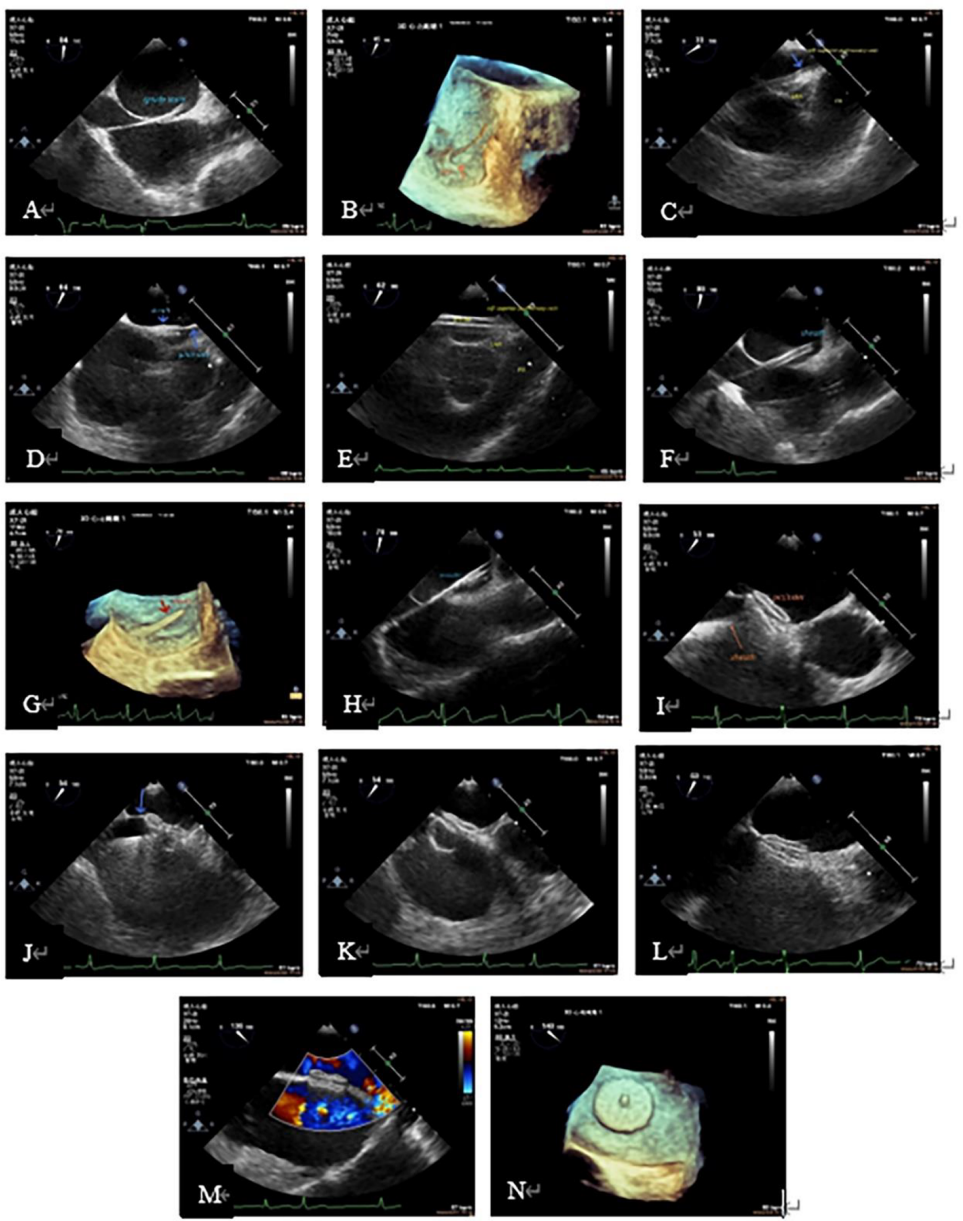
Procedure demonstration during TEE-guided percutaneous PFO closure. (**A**): Guide wire through PFO tunnel; (**B**): 3D visualization of the guidewire reaching the LA through the PFO. (**C**): Guidewire leading to LSPV (blue arrow); (**D**): the sheath (short blue arrow) passed through the PFO into the LA via the guide wire (long blue arrow). (**E**): Sheath entering the LSPV; (**F**): the sheath in the LA; (**G**): three-dimensional image showing the delivery sheath reaching the LA via the PFO; (**H**): occlude in the delivery sheath (echogenic part in the sheath); (**I**): after completely releasing of the two discs of the occlude, the atrial septum was located between the two discs, when the delivery sheath was still connected to the right atrial disc (arrow). (**J**): With the help of the sheath tube, the occlude was “pushed” to the LA, and the primary septum was pushed up (arrow). (**K**): The whole occlude was “pulled” to the RA, and the slight deformation of the right atrial disc could be seen. (**L**): State after the complete release of the occlude (complete detachment of the sheath from the right atrial disc). (**M**): Color Doppler showed no residual shunt. (**N**): Left atrial disc showed in 3D. 3D, three-dimensional; LA, left atrium; LSPV, left superior pulmonary vein; PFO, patent foramen ovale; TEE, transesophageal echocardiography.

All patients underwent TEE-guided percutaneous PFO closure under routine anesthesia in the operating room. The right femoral vein was the first choice to establish venous access. The whole TEE-guided procedure was divided into three steps:

(1) **Preoperative TEE** (Figure 1) A comprehensive preoperative TEE was necessary, including evaluations of cardiac function, the presence of pericardium, and most importantly, the exclusion of other cardiac conditions requiring surgical treatment. The observation content was the anatomical characteristics of the FO, including the size and length of the FO, the thickness and mobility of the primary septum, the presence of thrombosis in the FO, the size and direction of the FO shunt and the number of shunt tracts. In addition, it would be necessary to observe whether there was an atrial septal aneurysm (ASA) and excluded the presence of a thrombus within the aneurysm sac, the existence of a long Eustachian valve, and the presence of an atrial septal defect (ASD), which could provide reference for the selection of occlusion path, size and number of occluder.
(2) **Intraoperative guidance** (Figure 2): This is the main step. First of all, for patients without obvious shunt in preoperative TEE, we needed to further clarify the shunt before occlusion. At the view showing the “slit-like” channel of the FO, the head end of the delivery sheath was placed at the RA side of the FO, and 20 ml sterile saline was quickly injected. At this moment, the sonographer should observe the flow of the liquid. If it was shown in the LA with crystal reflection (appearance on ultrasound is a punctate hyperechoic), it indicated the existence of PFO. If not, the presence of a PFO was excluded.

This step is especially important for patients with a small gap: first, we can confirm the existence of the PFO, the size and length of the FO; second, we can confirm the path of the occlusion, which can reduce the procedure time, improve the occlusion efficiency and reduce complications. For patients with a clear gap and shunt, it is necessary to choose a view that can clearly show the gap of the FO (if there is a shunt, it is better to show the shunt and the gap together).

The procedure included the following steps: ① the guide wire was guided through the FO to the left superior pulmonary vein (LSPV). ② Guide the delivery sheath (if the gap is large enough) to follow the wire into the LSPV. If the gap is too small to directly pass through the delivery sheath, the inner core should be used for a certain auxiliary expansion. Because the tip of the inner core is sharp, the cooperation between the sonographer and the operator is very important during this process. The operator should push the inner core forward very slowly until the tip is exposed. This process does not need to expose the inner core head end completely: 5–10 mm in length is enough. Then, under the close observation of the sonographer, the whole delivery sheath was slowly advanced along the gap of the FO into the LA. The inner core was then returned to the sheath, and finally the delivery sheath was delivered to the LSPV. When the FO is too small, the guide wire occasionally accidentally breaks the secondary septum and enters under the capsule of the left atrial surface of the secondary septum when trying to enter the gap. At this time, it is difficult to identify whether the guide wire is in the channel of the FO or under the capsule, because the capsule is too thin to distinguish from the primary septum. In order to determine the position of the guide wire, we also took the method of injecting heparinized sterile saline (1,000 ml sterile saline + 100 mg heparin) through the delivery sheath. In contrast to the previous, this process requires a slow injection. Before this step, we withdrew the sheath to ensure that no gas remains inside (i.e., the evacuation process), thus avoiding the occurrence of air embolism. If the guide wire is in the FO channel, this process will be very smooth, and the crystals in the LA can be reflected at the same time. If not, there will be resistance during the injection process, and there is no crystal reflection in the LA. On the contrary, a raised capsular and a dark area of crystal reflection can be seen, which can become larger with the injection. In this case, the operator needs to withdraw the guide wire and re-adjust the access until it enters the correct FO gap. During this process, the method of injecting heparinized sterile saline can be used repeatedly to determine the position of the guide wire to ensure the safety and feasibility of the occlusion process. ③ Guide the release of the occluder: after the occluder was installed, the operator completely withdrew the inner core of the delivery sheath from the sheath, exhausted the sheath tube (exhaust is a very important step, otherwise coronary artery gas embolism is likely to occur), placed the occluder device in the delivery sheath, and slowly pushed it. When the occluder can be identified by the ultrasound screen, the sonographer should closely follow the position of the occluder. When the occluder came out of the sheath, the delivery sheath was withdrawn to the LA as a whole, and the operator synchronously slowly released the occluder until the left atrial disc was completely released. The transport sheath continued to withdraw. When the left atrial disc was close to the atrial septum, the right atrial disc was quickly and completely released. When this step was completely done, the delivery device was still connected to the right atrial disc. The sonographer needs to observe the position of the occluder, whether the two discs are on both sides of the atrial septum, whether there is residual shunt at the atrial level, and whether there is pericardial effusion. After confirming the two discs were completely separated and were located on either side of the atrial septum, thereby no residual shunt and pericardial effusion, the operator needed to conduct the “push” (push the occluder to the LA, if the atrial septum is shown to be jacked up, means in the correct position) and “pull” (pull the occluder backward to the RA; if the position is correct, it is visible that the disc is deformed by pulling) tests on the occluder to ensure the occluder is located on either side of the atrial septum. One should repeat the tests 2–3 times to ensure the stability of the occluder. After the completion of “push” and “pull” tests, the sonographer reconfirmed the position of the occluder and the shunt at the atrial level. If the occluder position is fixed and there is no shunt at the atrial level, the delivery sheath can be removed from the occluder device. The whole process of occlusion was completed. ④ After transcatheter closure, the main observations were whether the position of the occluder was fixed, and whether there was residual shunt at the atrial level, thrombosis, pericardial effusion and cardiac function.

### 2.4 Follow-up

All patient underwent a routine echocardiography examination before discharge. We conducted telephone follow-up or face-to-face follow-up at the patient's 1-month, 3-month, 6-month, 1-year, and 2-year postoperative visits. The follow-up included the clinical symptoms improved conditions, any thrombotic events happened, whether presented atrial fibrillation, whether there was residual shunting on echocardiography, and re-intervention.

### 2.5 Statistical analysis

Statistical analysis was performed using IBM SPSS software (version 26.0; IBM Corp.). Data was represented by (mean ± SD) for continuous variables and as frequency (*n*) and percentage (%) for categorical variables.

## 3 Results

**Figure 3:**
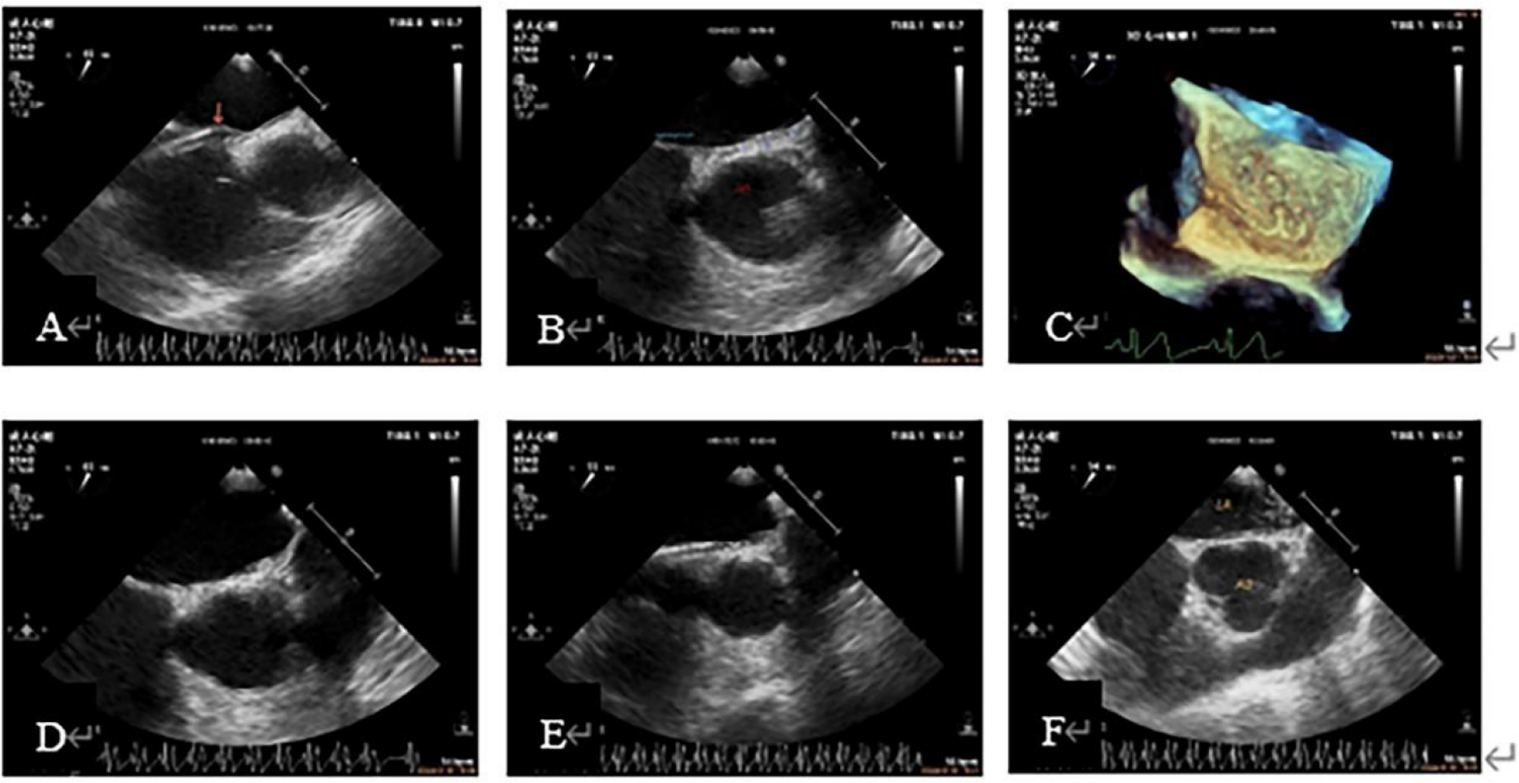
Case 1 A 53-year-old woman with migraine for more than 10 years had a grade 2 result by ASCE and preoperative TEE showed a PFO about 1 mm wide. (**A**): The head end of the delivery sheath was placed at the RA opening of the PFO, and the primary septum was pushed up (arrow). (**B**): Guidewire walking under the capsular of the secondary septum. (**C**): The tortuous guidewire shadow under the capsular of the secondary septum was shown in the 3D view of the LA surface. (**D**, **E**): The secondary septum was lifted after a slow injection of saline through the delivery sheath, and the hyperechoic subcapsule was observed, which proved that the sheath tube was under the secondary septum capsule and not in the LA. (**F**): Strong crystal echo in the LA after readjusting the path of the guidewire, indicating that the path was correct and the patient successfully completed PFO closure. 3D, three-dimensional; ASCE, agitated saline contrast echocardiography; LA, left atrial; RA, right atrial; PFO, patent foramen ovale; TEE, transesophageal echocardiography.

**Figure 4:**
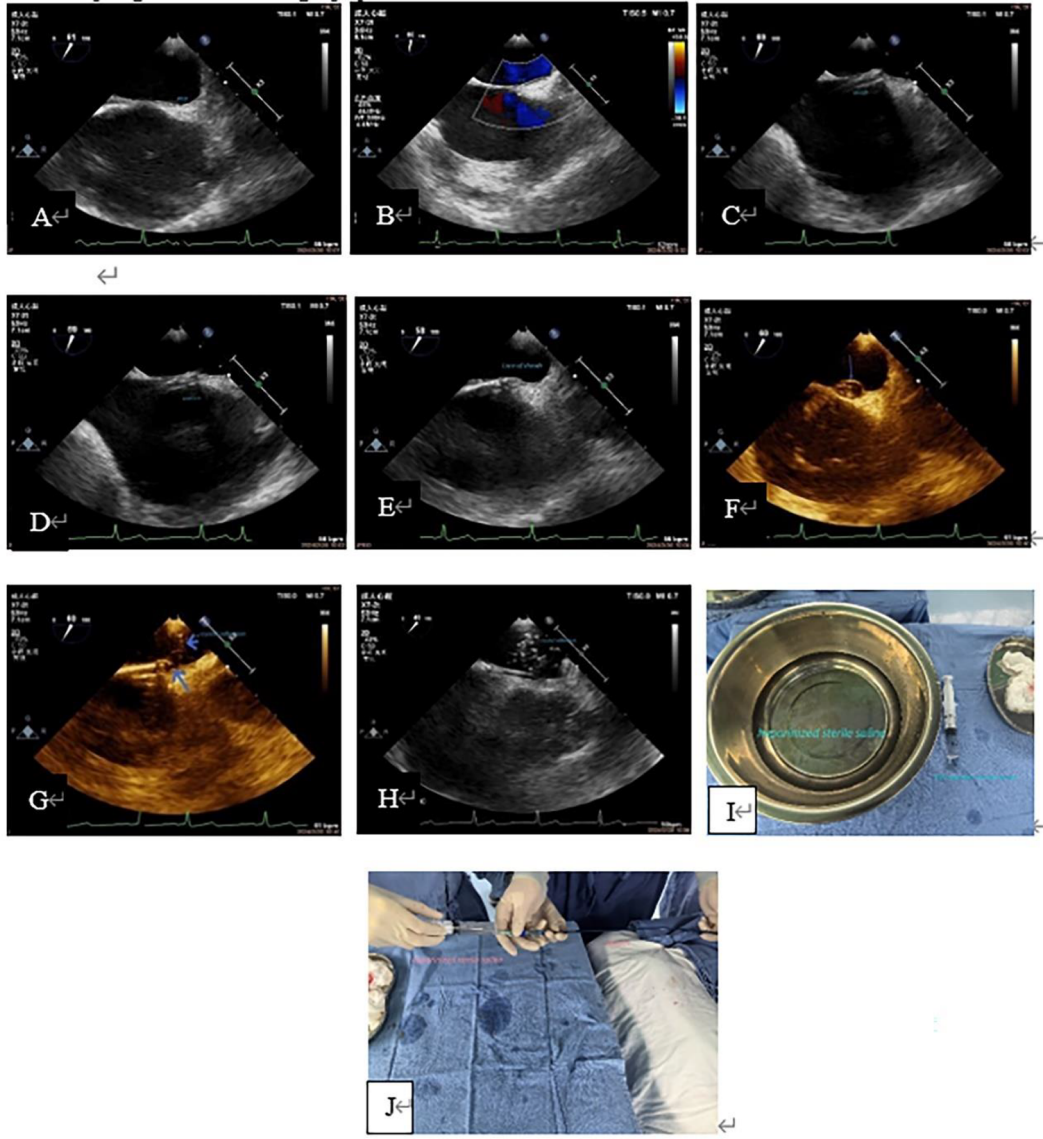
Case 2 A 31-year-old man with a history of cerebral infarction who had a positive result by ASCE (grade 3) and a 12 mm PFO tunnel width on preoperative TEE. (**A**, **B**): Preoperative TEE showed a small PFO channel and minimal LRS. (**C**): The delivery sheath was capped at the RA opening of the PFO. (**D**, **E**): The inner core was slowly sent into the PFO tunnel. (**F**): Saline was slowly injected through the sheath and the primary septum was lifted; the PFO appeared as a balloon (arrow). (**G**): Showed a pore in the primary septum and secondary septum (long blue arrow), and hyperechoic crystal was seen entering the LA through the pore (short blue arrow), which indicated the existence of PFO channel and correct delivery path of the sheath canal. H: showed that a large number of crystal echoes appear in the LA, indicating that the delivery sheath was entirely in the LA. (**I**): Showed the heparinized normal saline solution and a 20 ml syringe needed for the test. (**J**): Showed “injection of heparinized sterile saline through the delivery sheath”, this process was carried out jointly by nurses and doctors. This patient also underwent a successful occlusion. ASCE, agitated saline contrast echocardiography; LA, left atrium; LRS, left-to-right shunt; PFO, patent foramen ovale; TEE, transesophageal echocardiography.

3.1 **Baseline Information of patients** (Table 1): The average age of the subjects was 43.25 ± 14.87 years old. There were 45 females (61.64%), 29 patients with migraine (39.73%), 14 patients (19.19%) with headache and dizziness, 14 patients (19.18%) with a history of cerebral infarction, and 25 patients (34.25%) with cerebral infarction, lacunar infarction or ischemic focus on MRI. The duration of migraine and dizziness ranged from 1 month to 40 years. Among the subjects enrolled, 11 (15.07%) had a history of hypertension, 3 (4.11%) had a history of diabetes, and 4 (5.48%) had a history of coronary heart disease. There were 21 patients (28.77%) with PFO on TTE color Doppler, 66 patients (90.41%) with PFO confirmed by TEE before admission, and the average size of PFO was 1.48 ± 0.86 mm and the average length of PFO was 11.87 ± 5.44 mm. There were 16 cases (21.92%) of complex PFO. Preoperative ASCE showed that there were RLS, including 7 cases of grade 1 (9.59%), 12 cases of grade 2 (16.44%), and 35 cases of grade 3 (47.95%).
3.2 **“Injection of heparinized sterile saline through the delivery sheath” has a good value in assisting the diagnosis of PFO and confirming the closure path** (Figures 3, 4): Two cases were used to demonstrate the application of this method in path confirmation and PFO diagnosis.
3.3 All patients completed at least 3-months clinical and echocardiography follow-up, the longest follow-up being 2 years and 6 months (mean average 14 ± 10months; median time 14 months.). There were 2 patients with recurrent cerebral infarction at 1 month and 7 months post-operation respectively, and were relieved after corresponding treatment, and no embolic events occurred within 2-year follow-up. Among the patients with migraine and other symptoms, 3 patients felt that their symptoms did not improve at all after the operation, and their follow-up time was 3 months to 6 months. 11 patients had complete symptom relief, and 57 patients had symptom improvement. All patients had no atrial fibrillation after the operation, and no residual shunts or re-interventions were found on echocardiography follow-up (Table 2).

**Table 1:** Baseline data of patients undergoing percutaneous PFO closure. Grade 1 (few RLS): 1–10 MBs/frame in LA; Grade 2 (moderate RLS): 11–30 MBs/frame in LA; Grade 3 (massive RLS): >30 MBs/frame in the LA, or the LA was almost filled with MBs with significantly reduced sound transmission.

Patients’ baseline data ($\underline{\chi }$±s, *n* (%)		
Basic information		
Age (year)		43.25 ± 14.87
Female		45 (61.64)
Migraine		45 (61.65)
Dizziness		23 (31.51)
History of CI		14 (19.18)
HBP		11 (15.07)
DM		3 (4.11)
CHD		4 (5.48)
Auxiliary examination		
MRI		25 (34.25)
TTE		21 (28.77)
ASCE	Grade 1	7 (9.59)
	Grade 2	12 (16.44)
	Grade 3	35 (47.95)
TEE		66 (90.41)
Characteristics of PFO		
Size (mm)		1.48 ± 0.86
Length (mm)		11.87 ± 5.44
Complex PFO		16 (21.92)

ASA, atrial septal aneurysm; ASCE, agitated saline contrast echocardiography; CHD, coronary heart disease; CI, cerebral infarction; DM, diabetes mellitus; LA, left atrial; MB, microbubbles; MRI, magnetic resonance imaging; PFO, patent foramen ovale; RLS, right-to-left shunt; TEE, transesophageal echocardiography; TTE, transthoracic echocardiography.

**Table 2:** Short-term and middle-term follow-up. Symptom relief includes a decrease in the frequency and/or severity of symptoms.

		*n* = 73	
Complications of the procedure (case/%)	Anesthesia-related complications	0	0
	Pericardial effusion	1	1.37
	Vessel rupture	0	0
	Pericardial tamponade	0	0
	Hepatic vein injury	0	0
	Embolic events	0	0
Complications of the TEE (case/%)	Mucosal bleeding	3	4.11
	Esophageal and gastric perforation	0	
Follow-up (case/%)	Embolic events	2	2.74
	Symptom completely disappeared	11	15.07
	Symptom relief	57	78.82
	No improvement in the symptoms at all	3	4.11
	Atrial fibrillation	0	0
	Residual shunt	0	0
	Re-intervention	0	0

## 4 Discussion

In recent years, with the continuous improvement of interventional technology and biomaterials, the treatment of structural heart disease has gradually transformed from open surgery to interventional surgery. PFO, as a common disease, is closely related to a variety of clinical conditions. In 1992, Bridge et al. described the first transcatheter closure of PFO with the use of Bard Clamshell atrial septal umbrella after presumed retrograde embolization (7). Since then, the treatment of structural heart disease has entered a new era of intervention. The subsequent results of four randomized controlled trials in 2017 and 2018 (8–11) improved the management of PFO in patients with unexplained stroke/TIA, and substantially revised and updated the global guidelines (12, 13).

Migraine, a paroxysmal disorder characterized by complex sensory dysfunction and headache, is the second leading cause of disability worldwide (14). It is also a common disease in young people, and one-third of these patients have migraine aura (15, 16). Moreover, studies have proved that PFO is closely related to migraine (17), and migraine aura is related to RLS including PFO (18, 19). The mechanism of migraine caused by PFO is believed to be the transfer of vasoactive substances, which are usually filtered by the pulmonary circulation and enter the systemic circulation (18). Studies have shown that interventional closure of PFO can effectively reduce the frequency and duration of headache attacks compared to medications (4). And a large number of meta-analyses have shown that percutaneous PFO closure can effectively improve the duration and frequency of migraine symptoms in patients with migraine (20–23). Therefore, more and more migraine patients with PFO are suggested to choose percutaneous PFO closure as their treatment plans.

Cryptogenic stroke (CS) refers to stroke after exclusion of other identifiable stroke mechanisms, such as large-artery atherosclerotic disease, established cardiac embolic source, small-vessel occlusive disease (lacunar stroke), hypercoagulation disorder requiring anticoagulant therapy, or arterial dissection. Up to 40% of patients have unknown etiology (24), and PFO is present in 50% of CS patients <60 years old (25, 26), which is almost twice the prevalence in the general population (25). The mechanisms of CS caused by PFO include paradoxical thrombosis, *in situ* thrombosis, and arrhythmia (27), while paradoxical embolism is considered the most common mechanism, in which venous thrombosis enters the systemic circulation through the open PFO. There has been data suggesting that percutaneous PFO closure is superior to antiplatelet therapy in reducing the risk of recurrent stroke in selected patients under 60 years of age (8–11). And several meta-analyses also have confirmed that PFO closure reduced the risk of ischemic stroke in cryptogenic stroke patients with concomitant PFO (28, 29). According to the European Stroke Organization (ESO) guidelines, in adults under 60 years of age with cryptogenic stroke/transient ischemic attack and high-risk PFO features (moderate or severe shunt, ASA, atrial septal overactivity), percutaneous PFO closure plus medical therapy are recommended over antiplatelet therapy alone (30). Many clinical observations have confirmed PFO closure as a safe and effective treatment method to prevent recurrent cerebral embolism events (31, 32).

Since 1974, King and Mills performed the first successful percutaneous closure of ASD with the assistance of x-ray (33), tremendous intervention and innovation have occurred in the field of percutaneous

ASD closure using a transcatheter-based device during the last 70 years. Because closure devices for ASD can also be used for preventing paradoxical embolism in transcatheter closure of PFO, a subtype of secondary septal defect, percutaneous closure techniques have also been used in the treatment of PFO. Percutaneous PFO closure is similar to ASD closure except for the difference in occluder.

Although the guidance of x-ray can observe the whole process of delivery and occlusion, it also has its limitations including ① it is radioactive; ② it has certain risks of carcinogenesis, chromosome malformation and blood diseases; ③ lipiodol contrast agent also has a certain effect on the patient's body; ④ more importantly, the intracardiac structures and adjacent anatomical relationships could not be shown; ⑤ for some small PFO or complex PFO, the operation can not be performed or takes a long time, which will cause certain mental stress to the patients in the alert state. Meanwhile, due to the limitation of the fluoroscopy-guided PFO closure, the PFO cannot be further clarified during the procedure, and a comprehensive evaluation of the PFO cannot be performed, which also means that the appropriate occluder cannot be selected preoperatively. In addition, it is impossible to exclude whether there are small atrial septal defect (ASD) that cannot be identified in TTE examination, especially those that are very close to the PFO. It is impossible to identify and confirm whether exist thrombus in the PFO tunnel pre-operation and whether the closure device surface has thrombus post-operation. These reasons can easily lead to residual shunts and untreated atrial communication after the closure, thereby increasing the post-closure reintervention rate and also lead to thrombotic events during and after the procedure.

TEE has become an indispensable application in cardiac surgery (34, 35); especially, in valve surgery, it has become the standard intraoperative monitoring. Compared to only fluoroscopy-guided PFO closure, TEE not only improves the success rate of surgery (36), reduces the mortality of patients, but also reflects the hemodynamic changes in real-time. It is also increasingly used and plays an important role in coronary artery and other structural cardiac surgery, and is an important tool for intraoperative evaluation of regional ventricular wall motion or structural abnormalities (37). Percutaneous PFO closure has become the first choice for patients with indications due to its high success rate, minimal trauma and rapid postoperative recovery. TEE-guided PFO closure has the following characteristics and advantages: ① no radiation. ② Minimum injury: esophageal mucosal injury is the most common complication, but it usually recovers soon after esophageal probe evacuation. ③ Does not affect the surgical operation. ④ Real-time visualization: the TEE probe is located behind the LA, which allows close observation of the intracardiac structure. The anatomical characteristics of the PFO can be observed before the operation, and if necessary, the sterile saline injection test can be used to confirm whether an RLS is present or whether the intervention path is correct. It can provide more preoperative information and effective evidence for clinicians to make surgical decisions. In addition, it can assist the exhaust, closely observe the coronary artery gas embolism or not, dynamically observe the changes in cardiac function, and reduce the malignant complications such as cardiac arrest caused by coronary artery gas embolism. The position of the occluder, residual shunt and thrombosis could be observed after operation. ⑤ Less surgical complications and high safety: due to the real-time visualization of TEE, the incidence of cardiac injury or rupture and malignant complications such as cardiac arrest can be reduced. ⑥ Intraoperative “ heparinized sterile saline injection test” can improve the intraoperative diagnosis rate of PFO and the success rate of operation, and reduce surgical trauma. Clinically, there are quite a number of patients, including those with a history of stroke, whose primary septum is relatively thin, even though they show strong positive results in ASCE, but there is no obvious gap or shunt visible on TEE. TTE is more difficult to observe the gap and shunt in these patients, so we chose the method to further confirm the evidence of communication between the left and right atria during surgery.

In summary, TEE is essentially the operating surgeon's eyes. TEE can monitor the position, movement direction, and speed of the delivery device in the heart throughout the procedure. The serious complication of pericardial tamponade is mostly due to improper operation, such as excessive force applied by the operating surgeon, excessive speed, or heart ear rupture caused by these factors, under TEE guidance it can be prevented by providing timely feedback to the operating surgeon and communicate in real-time. During the procedure, we often encounter changes in the patient's heart rhythm, which are related to the operation, but with TEE, we can provide real-time feedback on the position of the delivery device, which can prevent improper operation from continuing and reduce or avoid the occurrence of serious complications.

## 5 Conclusions

TEE, a real-time visualization examination technology, has the characteristics of small injuries, no radiation, and no influence on surgical operation, which can reduce surgical complications and is highly safe. It has a high clinical application value in PFO closure. Intraoperative “ heparinized sterile saline injection test” can improve the intraoperative diagnosis rate of PFO and the success rate of operation, and reduce surgical trauma. This method is innovative, simple, and easy to operate and popularized in clinical practice.

## 6 Limitations

The main limitation of this study was not a randomized controlled trial, the sample size was not sufficient and the follow-up time was not long enough to provide more convincing evidences. Moreover percutaneous PFO closure was performed by different operators, due to their varying experience had different surgical techniques and operation times. Additionally, the choice of occluder by different patients based on their economic ability also varies.

TEE is to monitor the whole process of the operation by placing the probe in the middle of the esophagus and behind the LA, and it cannot observe the process of the guide wire and delivery sheath from the femoral vein to the right atrium, which may lead to: ① when the guide wire crosses the iliac vein confluence and enters the contralateral iliac vein, it cannot be detected in real-time. Its detection depends on the operators' and sonographers' experience; for instance, sonographers paying specific attention to the detection when not finding the guide wire in the RA; ②. The sharp inner core of the delivery sheath may damage the vessel wall or perforate during the push process, leading to retroperitoneal hematoma. In order to avoid this situation, our experience is that the delivery sheath with the inner core is pulled into the outer sheath about 2 cm after the femoral vein travels.

## Data Availability

The original contributions presented in the study are included in the article/Supplementary Material, further inquiries can be directed to the corresponding authors.
